# Why (we think) facilitation works: insights from organizational learning theory

**DOI:** 10.1186/s13012-015-0323-0

**Published:** 2015-10-06

**Authors:** Whitney Berta, Lisa Cranley, James W. Dearing, Elizabeth J. Dogherty, Janet E. Squires, Carole A. Estabrooks

**Affiliations:** 1 Institute of Health Policy, Management & Evaluation, Dalla Lana School of Public Health, University of Toronto, 155 College Street, 4th Floor, Toronto, Ontario M5T 3M6 Canada; 2 Lawrence S. Bloomberg Faculty of Nursing, University of Toronto, Toronto, Canada; 3 College for Communication Arts & Sciences, Michigan State University, East Lansing, Michigan USA; 4 St. Paul’s Hospital, Providence Health Care, Vancouver, British Columbia Canada; 5 School of Nursing, University of Ottawa, Ottawa, Canada; 6 Faculty of Nursing, University of Alberta, Edmonton, Alberta Canada

## Abstract

**Background:**

Facilitation is a guided interactional process that has been popularized in health care. Its popularity arises from its potential to support uptake and application of scientific knowledge that stands to improve clinical and managerial decision-making, practice, and ultimately patient outcomes and organizational performance. While this popular concept has garnered attention in health services research, we know that both the content of facilitation and its impact on knowledge implementation vary. The basis of this variation is poorly understood, and understanding is hampered by a lack of conceptual clarity.

**Discussion:**

In this paper, we argue that our understanding of facilitation and its effects is limited in part by a lack of clear theoretical grounding. We propose a theoretical home for facilitation in *organizational learning theory.* Referring to extant literature on facilitation and drawing on theoretical literature, we discuss the features of facilitation that suggest its role in contributing to learning capacity. We describe how facilitation may contribute to generating knowledge about the application of new scientific knowledge in health-care organizations.

**Summary:**

Facilitation’s promise, we suggest, lies in its potential to stimulate higher-order learning in organizations through experimenting with, generating learning about, and sustaining small-scale adaptations to organizational processes and work routines. The varied effectiveness of facilitation observed in the literature is associated with the presence or absence of factors known to influence organizational learning, since facilitation itself appears to act as a learning mechanism. We offer propositions regarding the relationships between facilitation processes and key organizational learning concepts that have the potential to guide future work to further our understanding of the role that facilitation plays in learning and knowledge generation.

**Electronic supplementary material:**

The online version of this article (doi:10.1186/s13012-015-0323-0) contains supplementary material, which is available to authorized users.

## Background

### The relevance of facilitation to knowledge implementation

Facilitation is both a *role* (a facilitator) and a *process* [1]. In the health-care sector, facilitation is championed as a mechanism to strengthen research utilization (the use of research-based scientific knowledge by practitioners) with the ultimate aims of improving health outcomes and organizational performance. Facilitation is effective in promoting research utilization in some, but not all, care settings [2, 3]. Our understanding of *how*, *why*, and *under what conditions* it is effective is generally poor. Dogherty et al. [1] note increasing calls to formally evaluate change initiatives that include facilitation. Challenges to understanding facilitation’s effectiveness, however, relate largely to persistent conceptual ambiguities. Facilitation [4, 5] and the facilitator’s role (see [6–8]) are conceptualized and operationalized inconsistently, and effectiveness is variously defined and measured. Consequently, we have little truly generalizable knowledge about how to construct facilitation processes to optimize research utilization, how to instruct the behaviours of facilitators, and how to appropriately set the degree of facilitation.

Organizational learning theory, we suggest, lends clarity to the concept of facilitation and offers explanations for its varied success. We see facilitation as similar to conceptualizations offered of *absorptive capacity meta-routines* [9, 10]. These are bundles of routines that are vital to an organization’s ability to acquire, apply, and learn adaptively about new knowledge to improve its performance [11]. Hence, we re-conceptualize facilitation as a meta-routine that specifically supports acquisition of and learning about applying research evidence to improve care processes.

### Organizational learning theory

Organizational learning theory is a meta-theory that considers the socio-organizational context of learning about new knowledge, the individual level factors that influence learning about new knowledge, the macro-environmental influences on knowledge application and learning, and the impact of the nature of the knowledge or innovation on subsequent learning processes [10, 12, 13]. This comprehensive theory is highly relevant to understanding knowledge translation phenomena [14].

Organizational learning is a social process. Members of an organization interact to construct meaning and knowledge about action-outcome relationships and about effects of the organization’s context (learning environment) on those relationships [15–19]. Some learning manifests as observable changes in worker behaviours and work routines. Other learning is not observable, such as learning that leads to decisions *not* to change. Individuals in organizations learn in a social context of other learners, with prior learning and accrued knowledge embedded in that context. Organizational learning therefore is more than the sum of what individuals know and learn, and it can persist well beyond the tenure of individuals. Learning that persists may be captured in explicit and encoded formal policies and procedures, in information and data collection systems [12, 20, 21], or in less explicit forms likened to *reservoirs* in an organization’s memory, informal communication channels, culture, and behavioural norms [15, 22].

#### The learning-performance link

Organizational learning is related to organizational performance [10]. An extensive empirical literature spanning diverse industries documents the positive effects on performance of *experiential learning*, which accrues as workers gain experience with repeated application of work routines [23]. Performance improvements are the products of adaptive learning, which arises through accrued experience and enables organizations to knowingly *adapt* their work routines [24]. Adaptive learning has been observed in health-care settings, for example, with the adoption of minimally invasive cardiac surgery procedures [25].

Adaptive learning can occur naturally and passively over time with the accrual of experience or it can be intentionally orchestrated. In the latter case, learning arises by introducing variation in *ways of doing* through importing new knowledge into an organization, often in the form of a pilot or a small-scale test. New knowledge that appears to resolve identified problems or affords the desired performance improvements is selected and retained. This cycle of *variation-selection-retention* is discussed by population ecologists [26, 27] as the chief means of evolution for whole “populations” of organizations over time. At the organization level and within its units or micro-systems [28], this cycle can be influenced or managed by astute organizational actors. Organizations that learn in this way are termed learning organizations.

While all organizations likely learn through accrual of experience, they do not all learn equally adeptly. Performance variation exists in every industry [12, 29], attributed in part to differences in *rates* at which organizations learn [12], *how* they learn [30], and the resources available for learning—their learning capacity.

#### Orders of organizational learning

To understand performance variation and its relationship to learning, adaptive learning theorists [17, 30] distinguish three types of organizational learning. *Single-loop organizational learning* describes corrective actions in response to performance failures that focus exclusively on improving efficiency of existing routines or processes. Original routines are largely preserved along with the goals and values they were designed to achieve [31]. This is by far the dominant mode of learning in organizations. When organizations operate in particularly stable, unchanging environments, this mode is perfectly appropriate and incremental changes to routine production processes may improve efficiency.

In other situations, organizational actors respond to errors or performance failures by questioning the initial goals, assumptions, and values that led to a particular workplace process. The consequence of this questioning is *double-loop learning* that connects “understanding, insight and explanation to action” ([31], p. 1179). Double-loop learning may manifest as significant adaptive changes to workplace behaviours and routines and to goals, assumptions, and underlying values. The ability to engage in higher-order, double-loop learning is thought to be advantageous—if not vital [32]—to organizations operating in volatile, uncertain environments such as health care [33]. Changeable environments are thought to favour adaptive learners. Argyris and Schön [30] contend that engaging people in higher-order learning is important to exercise adaptive learning potential and equips organizations to perform closer to their aspiration levels.

**Fig. 1:**
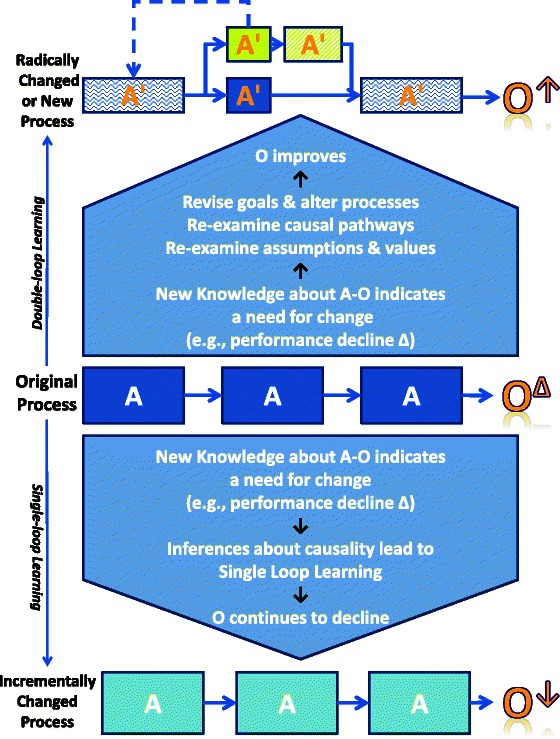
Single-loop and double-loop learning under conditions of high environmental uncertainty. *A* Actions are linked by *arrows* to comprise a process. *O* outcome(s) of a process

Figure 1 illustrates the distinctions between single-loop and double-loop learning under conditions of high environmental uncertainty, where the sequential actions (A) originally established to produce outcomes (O) of an “original process” are suggested as no longer ideal due to changes in the outcome (O^∆^). The original process may be replaced, as a consequence of double-loop learning (top of Fig. 1), with a radically changed or new process comprising a new set of actions (A′) linked in a new series or sequence and affording improved outcomes (O↑). When the response to new knowledge about A-O relationships leads only to single-loop learning (bottom of Fig. 1), processes incorporating only incremental changes resulting in essentially unchanged actions (A) performed in the same sequence will likely produce increasingly poor outputs (O↓) whether reflected as reduced output quantity (efficiency declines) or reduced quality (reductions in effectiveness).

The highest order of learning is *triple-loop organizational learning* (meta-learning or learning about learning) which refers to reflective learning about how and when learning does, or does not, occur [17]. Triple-loop learners focus on learning that improves their learning processes, in addition to adaptive learning that improves production processes and optimizes behaviours.

One feature that likely distinguishes high-performing organizations from poor performers, in uncertain environments like health care, is their capacity to engage their workers in higher-order learning. Higher-order learners, particularly triple-loop learners, will experience few limits to understanding action-outcome relationships and will be adept adaptive learners. We know from the literature that higher-order learning is rare relative to single-loop learning, as are top-performing organizations, not coincidentally. In sum, what we know about learning-performance links underscores the value of efforts to better understand learning capacity—what it comprises, and how and why some organizations can acquire or build it while others cannot or do not.

#### What we know about organizational learning capacity and learning processes

Learning capacity, also referred to as adaptive capacity or absorptive capacity, reflects an organization’s ability to recognize the value of new knowledge and information, assimilate it, and apply it to make high-quality decisions [9, 11, 34]. Research demonstrates learning capacity’s role in innovation and business performance [35], intra-organizational knowledge transfer [36, 37], and inter-organizational learning [38, 39]. Absorptive capacity is noted in the health services literature as relevant to an organization’s ability to effect performance improvements [40], assimilate innovations [41], and apply new knowledge [14].

Learning theorists conceptualize absorptive capacity as both a *precondition* to organizational learning and an *outcome* of it; Cohen and Levinthal [11] distinguish between potential absorptive capacity and realized capacity. Lewin et al. [9] usefully extend earlier discussions to conceptualize absorptive capacity, within an adaptive learning context, as composed of *external* and *internal* absorptive capacity meta-routines. *External absorptive capacity* refers to meta-routines (bundles of routines, processes, or activities) that an organization applies to exploring or scanning its external environment to discover new knowledge that might benefit it generally (proactive scanning) or to solve an existing performance problem (reactive scanning). Exercising external absorptive capacity introduces variation in an organization’s routines, as in the adaptive learning cycle of variation-selection-retention. Specific examples of routines and mechanisms indicating external absorptive capacity include the following: (1) situating dedicated organization resources at the organization’s boundaries (e.g., knowledge brokers [42], innovation offices, and strategic management functions) to identify and secure new outside knowledge with potential to solve organizational problems or enhance performance [11]; (2) establishing networks or collaboratives to engage with other industry actors (partners, suppliers, customers, competitors, and consultants) who can provide the organization with new operationally valuable knowledge [43]; and (3) establishing structural mechanisms like subject matter experts who take external knowledge brought to the organization’s boundary and ensure that it is shared, disseminated, or acted upon within the organization [9].

*Internal absorptive capacity* refers to meta-routines invoked once new knowledge is imported into an organization. Some meta-routines, like brainstorming or offering time and space for informal interactions, pave the way for change and facilitate internal variation [9]. Techniques founded on scientific management principles, like lean manufacturing, are also sources of variation that generate new knowledge about how to improve work processes. Pilot studies or organized experiments (Ng S, Berta W, Barnsley J. Realizing the adaptive potential of evidence-based knowledge: how, what & why learning occurs (or does not) during clinical practice guideline implementation: a multiple case study. Unpublished.) are routines that inform internal selection among alternative change initiatives. Routines including experiential training opportunities promote reflection and updating, while procedures like results reporting may also prompt replication [9]. Learning retention is another aspect of internal absorptive capacity. Routines relating to embedding or routinizing (sustaining) changes to work practices in the larger organizational context, or in micro-systems [44], are important. Equally important may be routines that facilitate replacement of existing routines and unlearning old ways of doing [23].

In health care, examples of routines relevant to internal absorptive capacity include pilot studies, cross-functional teams, within-organization formal and informal communication mechanisms, quality improvement initiatives, clinical and management information systems, and benchmarking.

**Fig. 2:**
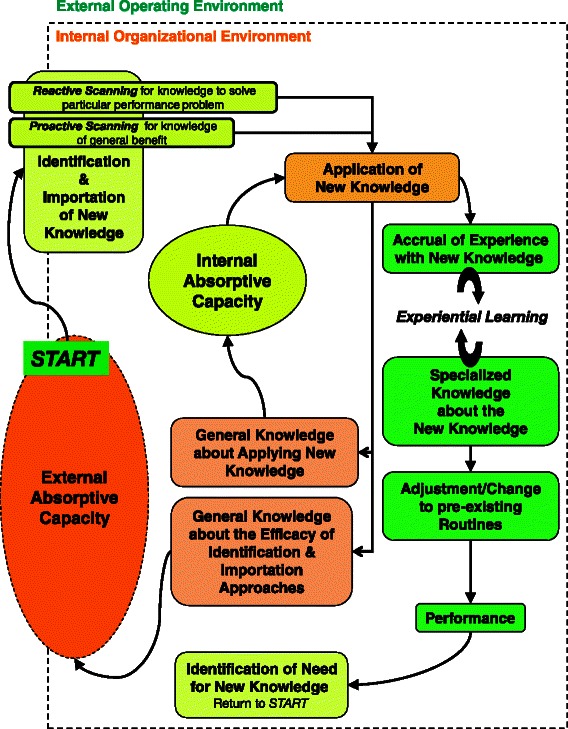
Relating concepts of knowledge implementation and external and internal absorptive capacity

Figure 2 illustrates roles of external and internal absorptive capacity meta-routines in knowledge implementation and social learning processes. External absorptive capacity meta-routines (enacted at the boundary between the depicted organization’s internal and external environments) detect and select new knowledge to import into an organization. Internal absorptive capacity meta-routines then apply that knowledge in situ and produce new knowledge *about* the new knowledge and its association with outcomes of interest to the organization*.*

We argue below that elements of *facilitation,* as conceptualized in the health care literature, serve as meta-routines that support higher-order social learning about new evidence-based knowledge. At the boundaries of organizations, some facilitation elements constitute external absorptive capacity meta-routines, while elements enacted within the organization contribute to internal absorptive capacity [45, 46]. Further, we conceptualize the role of a *facilitator* as a social integration mechanism that combats an organization’s tendency to lower-order learning.

#### Insights into organizational learning micro-processes from scientific management

While the organizational learning literature offers insights into learning meta-routines, it is bereft of insights into learning micro-processes. This omission has been remarked upon for decades [9]. Proxy measures of absorptive capacity are criticized for not helping our understanding of “specific routines or processes that constitute absorptive capacity and distinguish between the absorptive capacity capabilities of different organizations” ([29], p. 237).

While they not typically expressed in terms of learning and capacity, the scientific management literature [47] offers a number of insights on how organizations acquire adaptive learning capacity through concerted learning micro-processes. Quality or process improvement initiatives generally involve an array of techniques designed to create, and put into practice, socio-technical systems that integrate learning in organizations and enhance knowledge management [48]. As in other industries, scientific management techniques applied in health care generally target frontline workers and promote team-based approaches to solving problems, integrative learning, and effect process improvement. Many of these techniques are now widely diffused in health care and include lean manufacturing (derived from Toyota’s production system, emphasizing efficiency by reducing waste and redundancy), Six Sigma (developed by Motorola to enhance quality by reducing errors and defects), the Institute for Healthcare Improvement’s Plan-Do-Study-Act (PDSA) model, continuous quality improvement, and rapid cycle improvement. All of these techniques are intended to lead to manageable and replicable processes that capture what is learned about work, potentially culminating in the ideal of a learning health-care system [49] that maximizes quality, safety, service, and affordability, and in many of them, facilitation is a major component. As in other contexts, facilitation assists in defining practice problems and objectives, provides support to teams in achieving objectives, highlights important contextual factors, and assists teams in interpreting data and reaching conclusions about action-outcome relationships. In health care, these quality improvement initiatives often engage frontline workers, who have rarely or never been engaged before, in team-based problem-solving. By doing so, facilitation not only also serves to create new or strengthen existing relationships among workers but generates or renews the confidence and commitment among frontline team members and frees them to think in a different way about workplace problems, including questioning the underlying values and assumptions of the processes they are trying to improve. One hallmark of facilitation-based quality improvement initiatives is encouraging teams to see problems in their work contexts as things that they can affect and modify, rather than “just put up with.” While the focus is on monitoring and evaluation, the aim is on efficiency and perfecting processes, thus teams likely tend to undertake single-loop rather than higher-order learning.

## Discussion

### Facilitation defined and redefined as meta-routines

Here, we extend our description of facilitation and describe work in health care that highlights its intended function in organizational practice and performance improvement initiatives. We then describe linkages between the micro-processes generally associated with facilitation and the tenets of organizational learning theory discussed above and offer a series of propositions focussed on these relationships.

#### Elements of facilitation

In the health services literature, facilitation is a concerted, social process that focuses on evidence-informed practice change and incorporates aspects of project management, leadership, relationship building, and communication. It has three main components: (1) the facilitator role and associated activities, (2) facilitation processes and essential components, and (3) outcomes of facilitation.

A *facilitator* is someone who acts and enables others to implement a practice change. The role may be internal or external (or both) to the organization implementing the change (e.g., [4, 6, 45]). The role of the facilitator is to help and enable rather than to prescribe [4]. Not all facilitators are formally trained for the role, while some receive extensive skills and development training [50–52].

One main activity of the facilitator is to encourage others to reflect upon their current practices in order to understand gaps in performance and where changes can be made (e.g., [6, 53–55]). This includes encouraging reflection on current attitudes, assumptions, and ways of working and identifying concerns, all aiming to enhance receptivity to change [56, 57]. The facilitator provides ongoing support tailored to local needs and circumstances, through activities that include introducing new ideas for change based on the identified need for improvement [55, 58], removing barriers and providing resources to assist with change [59–61], establishing effective communication channels among those making the change [62–64], and monitoring progress [1, 65]. An effective facilitator influences local climate and promotes a culture for change [66, 67].

Approaches to facilitation necessarily vary, but most have common features. Facilitation drives a purposeful, progressive, or iterative two-way process of change that focuses on building trusting relationships and establishing and sharing common goals between the facilitator and those engaged in making the change [55, 68]. Facilitation is a social process and takes a team-based approach to implementing change, generally through newly formed teams initiated by the facilitator. Critical elements driving successful facilitation are effective communication, interactive problem solving, and relationship building [1].

Dogherty and colleagues [1] identified four stages in facilitation-assisted implementation evidence: planning for change, leading and managing change, monitoring progress and ongoing implementation, and evaluating change (see also [45]). *Planning for change* involves increasing staffs’ awareness of a need for change and assisting with developing a plan for implementation. *Leading and managing change* includes fostering team building and group dynamics and providing project-specific support such as resources and tools for change. In *monitoring progress and ongoing implementation*, facilitators assist with problem-solving and provide ongoing support. *Evaluating change* involves conducting or assisting with performance evaluation and linking evidence implementation to patient outcomes.

Facilitation is typically initiated at the micro-system level of an organization. In health-care organizations, clinical micro-systems like patient care units form the frontline of care delivery [69, 70]. Clinical micro-systems are a central element of the quality improvement literature, emphasizing the importance of directing system improvement strategies at clinical (patient or resident care) units rather than the macro-organizational level. The greatest improvement and change can be made at the unit level [71] with small groups of people routinely working together to provide patient care [28]. New knowledge is often piloted at the clinical micro-system level prior to implementation across an organization.

The overall aim of facilitation is to support a sustainable evidence-informed practice change, based on an identified performance gap, which improves patient outcomes. We focus on *research evidence,* but the term *evidence* extends beyond research evidence (and beyond level 1 research evidence) to include case studies, expert or consensus opinion, or accumulated experience. All are valuable forms of evidence that can be brought to solving clinical problems or improving health-care performance and outcomes.

*Expected outcomes* of facilitation occur at three levels: individual, micro-system, and organizational. Individual level outcomes include changes to individuals’ thinking or ways of working. Micro-system level outcomes are changes in the ways individuals *work together* (e.g., at the team level). At the organizational level, facilitators help build infrastructures or meta-support (e.g., organizational systems and culture) necessary to underpin the success of innovations [4, 58, 68]. These organizational systems are generally characterized as organizational contexts supportive of change and evidence-based practice.

*Evaluating the success* or effectiveness of facilitation is also part of implementation and may involve ongoing monitoring of both process and outcomes [1]. Facilitation is expected to impart embedded and sustained practice change. We know that this requires continuous, collective investment by those making the change [44], but we do not know precisely which facilitation approaches, and which structures and processes, must be in place to maintain practice changes. These may be different within different contexts [1].

The many definitions of facilitation offered in the literature do not vary radically (see Additional file 1: Table S1). Based upon our review of the facilitation literature above and recent systematic reviews of the literature [1, 46], we offer this definition:

Facilitation is a goal-oriented, context-dependent social process for implementing new knowledge into practice or organizational routines. It typically involves individuals learning together in the context of a recognized need for improvement and supportive relationships. Effective communication and interactive problem solving are key process components.

### Situating facilitation theoretically in organizational learning

Here, we argue for situating facilitation theoretically within organizational learning theory, offering ten propositions on the relationships between facilitation processes and key organizational learning concepts. These propositions suggest the important and nuanced role of facilitation in contributing to external and internal absorptive capacity and to organizational learning and knowledge generation.

#### Facilitation’s role in realizing potential absorptive capacity

First, we contend that facilitation belongs to the set of social integration mechanisms referred to generically by Lewin et al. [9] as important to realizing absorptive capacity, and to understanding and leveraging power relationships and associated social dynamics in organizations. In health care, facilitation empowers staff closest to care processes to change care practice. These staff members are often underutilized in identifying work problems and improvement efforts. Facilitation equips staff with the skills and self-efficacy to act in resolving problems, by accessing and leveraging their potential or latent absorptive capacity at the unit level. Benefits may extend beyond the unit if intra- or inter-organizational sharing leads to adoption and productive adaptations by other units [72].

- *Proposition 1*: Facilitation is a social integration mechanism that leads to realizing (latent) absorptive capacity potential in health services organizations.

#### Linking facilitation to the meta-process of learning

At the level of the organization or unit, facilitation likely contributes to each component of the meta-process of variation-selection-retention that leads to adaptive learning (discussed in “The learning–performance link” subsection above). In facilitated settings, organizational actors are urged to reflect critically on how their work behaviours influence work performance. Facilitators assist them in identifying areas needing improvement. In this way, staff can become receptive to *variation*: in the form of new research-based ideas as potential solutions to identified needs or performance problems. Common goals are established that guide *selection* of a solution from among these idea alternatives. Social decision-making processes are foundational to facilitation and assist selection. Facilitation also strengthens *retention* of changes as it identifies and secures needed implementation resources, leverages communication channels, and establishes evaluation mechanisms.

- *Proposition 2*: Facilitation influences the learning meta-process of variation-selection-retention of new knowledge.

#### Relating facilitation to external and internal absorptive capacity meta-routines and organizational learning

The scope of facilitation micro-processes observed in health-care settings varies [46]. In some organizations, facilitation is an external absorptive capacity meta-routine while in others, it is largely internally focused. A few organizations with large facilitation initiatives may focus on both internal and external capacities. Situating facilitation appropriately in extant theory will advance our understanding of the mechanisms by which it influences the uptake and application of new knowledge (e.g., research knowledge), will enhance our ability to evaluate it, and will suggest meaningful future research. Micro-processes and activities identified with facilitation can be mapped to the *external* (Table 1) and *internal* (Table 2) absorptive capacity meta-routines discussed by Lewin et al. [9].

**Table 1:** Map of facilitation processes and activities to external absorptive capacity meta-routines

External absorptive capacity meta-routines [9]	Facilitation processes and activities^a^ [1, 46]
Identifying and recognizing the value of externally generated knowledge	Introduces new research-based ideas of potential value to resolving performance gaps
Learning from and with partners, suppliers, customers, competitors, and consultants	Establishes effective communication channels
	Networking
	Supports the development of new competencies or skills by identifying external suppliers
Transferring knowledge back to the organization (establishing knowledge sharing processes)	Establishes effective communication channels

^a^For primary sources, see Additional file 1: Table S1a

**Table 2:** Map of facilitation processes and activities to internal absorptive capacity meta-routines

Internal absorptive capacity meta-routines [9]	Facilitation processes and activities^a^ [1, 46]
Facilitating variation	Encourages critical assessment of current practice that leads to identification of performance gap(s)
	Introduces new ideas (i.e., research and associated knowledge that may address performance gaps)
	Enhances staff receptivity to change
	Identifies resources needed to support change
	Motivates and encourages others to make a change
	Supports the development of new competencies/skills among staff
Managing internal selection regimes	Assists in establishing common goals
	Enables implementation of evidence into practice
	Enables research use
	Attends to the process of achieving goals
	Provides feedback about research use
Sharing knowledge and superior practices across the organization	Establishes effective (internal) communication channels
	Promotes a culture for change
	Creates a supportive (local) climate
	Creates a vision that embraces evidence-based practice
Reflecting, updating, and replicating (retention)	Tailors facilitation activities to local needs and circumstances
	Provides ongoing support and resources to achieve goals
	Facilitates trialable initiatives
	Maintains change momentum
	Supports the development of new competencies/skills among staff
	Supports a goal-oriented dynamic process that promotes learning through critical reflection
Managing adaptive tension (continuous progression)	Creates a vision that embraces evidence-based practice
	Promotes a culture for change
	Creates a supportive (local) climate
	Empowers staff

^a^For primary sources, see Additional file 1: Table S2a

- *Proposition 3*: Facilitation micro-processes and activities (introducing new ideas, establishing effective communication channels, engaging in networking, and identifying suppliers of new competencies and skills) comprise external absorptive capacity meta-routines.

Of particular note, the facilitation processes mapped to external absorptive capacity in Table 1 are likely precursors to higher-order organizational learning. If new knowledge imported to the organization boundary through facilitation is ultimately applied within the organization, change may be needed to existing work routines, practices, and structures.

The foundational adaptive learning process of variation-selection-retention is represented among the meta-routines in Table 2. The facilitation processes and activities noted in the facilitation literature map readily to these meta-routines.

The propositions below state these linkages and offer a more granular complement to Proposition 2.

- *Proposition 4*: Facilitation micro-processes and activities (encouraging assessment of current practice, introducing new ideas, enhancing staff receptivity to change and encouraging or motivating them to make change, identifying resources for change, motivating others to make a change, introducing new ideas internal to the organization, and supporting development of new staff competencies and skills) comprise internal absorptive capacity meta-routines for *facilitating variation*.
- *Proposition 5*: Facilitation micro-processes and activities (assisting in establishing common goals, enabling the implementation of research findings into practice, attending to the process of achieving goals, and providing feedback about research use) comprise internal absorptive capacity meta-routines for *managing internal selection regimes*.
- *Proposition 6*: Facilitation micro-processes and activities (establishing effective communication channels, empowering staff, promoting positive changes in culture or climate, and creating a vision that embraces evidence-based practice) comprise internal absorptive capacity meta-routines for *sharing knowledge and superior practices across the organization*.
- *Proposition 7*: Facilitation micro-processes and activities (tailoring facilitation activities to local needs and circumstances, providing ongoing support and resources to achieve goals, facilitating trialable initiatives, maintaining change momentum, supporting development of new competencies and skills, and supporting a goal-oriented dynamic process that promotes learning through critical reflection) comprise internal absorptive capacity meta-routines for *reflecting, updating, and replicating*.
- *Proposition 8*: Facilitation micro-processes and activities (creating a vision that embraces evidence-based practice, promoting a culture for positive change, creating a supportive local climate) comprise internal absorptive capacity meta-routines for *managing adaptive tension*.

In facilitation, reflection leads to critical questioning of both work processes and the social structures that sustain practices and behaviours. An expected outcome of facilitation is structuring new ways of working and communicating, implicitly abandoning old, moderately effective, or ineffective structures. These activities reflect higher-order learning since they necessitate responding, through substantive practice and behaviour change, to information gleaned through critical reflection about action-outcome relationships. Many of the activities and processes undertaken by facilitators described in Table 2 have potential to foment higher-order learning. With the caveat that staff must have the capacity and willingness to learn, the learning enabled by facilitation is likely to take the form of double-loop learning. The critical reflection [73] demanded by facilitation leads to better understanding of action-outcome relationships, and consequent changes in worker attitudes, behaviours, and ways of doing [4].

- *Proposition 9*: Facilitation micro-processes and activities that relate to the internal absorptive capacity meta-routines of reflecting, updating and replicating can lead to higher-order, double-loop organizational learning.

The facilitation micro-processes mapped to reflecting, updating and replicating, and managing adaptive tension relate additionally to triple-loop learning. Further reflection may calibrate facilitation activities to the local context and to ways in which learning takes place (learning about learning). This could provide the environment, resources, and skills that contribute to and sustain informed continuous change.

- *Proposition 10*: Facilitation micro-processes and activities that relate to the internal absorptive capacity meta-routines of reflecting, updating, and replicating and managing adaptive tension can lead to triple-loop learning.

### Summary

An extensive literature focuses on facilitation in health care and its role in effecting positive practice change founded on research evidence. Numerous knowledge translation researchers have promoted facilitation as a mechanism to enhance uptake and application of research [4, 74], but the literature on effectiveness of facilitation in actually improving uptake is sparse and inconsistent [1].

In this paper, we attempt to ground existing facilitation literature in organizational learning theory. We contend that the value of facilitation as an organizational process that improves performance, and as a useful theoretical construct, lies in its potential to stimulate higher-order learning in organizations. Facilitation stimulates this learning by enacting micro-processes and activities that access, capitalize upon, and build internal and external absorptive capacity in organizations.

We offer ten propositions that explicate mappings between facilitation micro-processes and key organizational learning concepts. Future work to explore these propositions will contribute a deeper, more nuanced understanding of facilitation’s role in research implementation and in generating learning and knowledge associated with introducing new scientific evidence. Work of this nature would go some way toward realizing the promise of scientific evidence for improving clinical and managerial practices and patient outcomes and organizational performance. Our work has theoretical, practical, and policy implications.

#### Theoretical implications

Our work informs organizational learning theory in addition to assisting our understanding of facilitation’s role in generating learning and knowledge and offering insights into how this occurs. Exploring our ten propositions will enhance understanding of the micro-learning processes associated with higher-order learning and realizing the adaptive potential of new knowledge generated through learning [12, 30, 75]. This exploration will afford insights into the factors—contextual, evidential, or otherwise—that influence these processes [76] and will address the current absence of research into learning micro-processes that contribute to absorptive capacity [9, 29] and differentiate good organizational learners from poor learners.

Our work also responds to concerns around variation in conceptualizing facilitation, its effectiveness, and the role of facilitators [4, 5, 8]. This variation has frustrated efforts to evaluate facilitation and its effects systematically. Situating facilitation in organizational learning theory has implications for evaluating initiatives that incorporate facilitation as a mechanism to support uptake of research evidence. Facilitation effectiveness measures—none of which currently exist—might incorporate items, for example, that focus on the extent to which variation, selection, and retention are enhanced. Numerous measures are implied by the processes and activities in Tables 1 and 2. Effectiveness measures might also capture the extent to which facilitation enhances understanding of action-outcome relationships through agree-disagree statements like “I understand how what I do impacts my patients.” Other measures of facilitation effectiveness are the extent to which internal and external absorptive capacities increase with introduction of a facilitation-based quality improvement intervention and the extent to which higher-order learning occurs. For example, agree-disagree statements might detect double-loop learning such as “When we receive negative performance feedback, we revisit our assumptions about how what we do impacts our patients…” or “We make changes to our goals…policies…for patient care based on performance feedback.” Triple-loop learning might be assessed via agree-disagree statements like “Performance feedback led us to change the ways that we learn about our actions and their impacts on patients (e.g., create a standing Quality Improvement committee).”

Our discussion also resonates with other social theories that explain behaviour change. In particular, the linkages that we make here among organizational learning theory concepts—particularly higher-order learning, and its inherent challenges and facilitation are consistent with work that applies social practice theory [77] to explain behaviour change. Social practice theory, inspired in part by Giddens [78] structuration theory, involves the analysis of “practices” in social settings (including but not exclusive to organizational settings) that are both generated and sustained by shared understandings about the skills and knowledge required to complete activities, and these shared understandings are in turn shaped by assumptions and presuppositions [77] about what is referred to in the learning literature as action-outcome relationships [15]. When it comes to changing practices, practice theorists like Røpke ([79], p. 2492) underscore the importance of reflection—one key aspect of facilitation that we discuss above—which “opens actors to question the bases for their actions”—that is, the assumptions and presuppositions discussed in social practice theory and the action-outcome relationships discussed in organizational learning theory. Structure-actor dualism is prominent in social practice theory and is relevant to our discussion of practice improvement and change in the context of health care; while non-trivial changes to practices are likely to lead to changes in the social structure in health services organizations, we note that facilitation itself represents a structural perturbation, which leads in turn to changes in practices by requiring reflection, querying action-outcome assumptions, re-examination of goals and the knowledge and skills required to achieve them, and higher-order learning.

These new structures to support new practices are often hard won, particularly since both they and the new actions/practices that they support often replace or supplant existing structures and practices. Indeed, Hargreaves [79] refers to the intractableness of social structures, where practitioner-members must be persuaded to “defect” to alternate practices. In organizational learning theory, there is similar discussion of “reversion to old routines” [12] and the difficulties inherent in “unlearning” in order to learn new practices/routines [23]—at times, seemingly requiring something akin to an organizational revolution—that are phenomena that learning theorists relate as much to the constraints of material structure and inertia of social relations in organizations as they do to the attitudes and behaviours of individual organizational members. Finally, social learning theorists would likely also assert that facilitation and reflection importantly leads to querying the relevance of “material artefacts” to action-outcome relationships—that is, the equipment, tools, materials and infrastructure traditionally used in undertaking an activity [80].

#### Practical implications

Managers and facilitators in organizations planning evidence-based practice changes would benefit from orientation to the tenets of organizational learning and their relationships to facilitation micro-processes and activities. Our work suggests that initiatives incorporating elements of facilitation are more likely to benefit from adaptive, higher-order learning by staff that leads to positive practice or process change. This is thought to be the only type of learning that leads to sustained behavioural change in organizations [30]. Finally, adaptive knowledge generated through facilitation within a clinical micro-system might lead to widespread improvements in organizational process, if mechanisms are in place to disseminate knowledge intra-organizationally [72].

With its emphasis on adaptive learning capacity, the organizational learning literature underscores the importance of context to generating learning and knowledge. Managers should know that, in knowledge-intensive organizations and industries, much of this context is situated with organizational actors who are frontline workers, but it extends beyond frontline workers to include middle managers and senior leadership. The organizational learning literature suggests that earnest engagement of the capacity represented by these individuals, in addition to frontline workers, is likely to enhance an organization’s ability to learn adaptively and engage in higher-order learning. We note that many quality process improvement initiatives do not capitalize on this source of potential absorptive capacity, generally under-engaging workers at mid-levels in organizations and inadequately engaging workers at the frontline [81].

We note that health services organizations are likely to be much like other organizations in other sectors where most, at best, engage in single loop learning and peripheral change. We know from public reporting systems that there is considerable variation in performance among health services organizations, and we know from the literature that higher-order learning is rare relative to single-loop learning—as are high-performing organizations. Formidable challenges to change and organizational learning have been noted previously by health services researchers [20, 82], and we by no means intend to underplay the difficulties inherent in implementing practice change. That said, our discussion above highlights facilitation’s potential as a powerful social integration mechanism for realizing, and generating, absorptive capacity in health services organizations and fomenting sustainable practice change.

#### Policy implications

The Institute of Medicine has long invested resources to cultivate continuously learning health systems and has championed science-driven health care as the chief means of enhancing the industry’s performance [49, 83]. Learning health systems are those in which (in the language of organizational learning theory) higher-order learning meta-routines are embedded and adaptive learning is the norm. Facilitation that includes micro-processes relating to *external absorptive capacity* is important to identify scientific knowledge that can be applied to improve performance and support its transfer into organizations. Facilitation further appears to be a promising means to initiate workers into learning micro-processes that contribute to *internal absorptive capacity*, which supports higher-order learning and innovation. We emphasize the value of facilitation in capitalizing on the performance potential of workers—particularly frontline workers who are typically under-engaged in performance improvement initiatives—and on scientific knowledge (e.g., research evidence) to inform positive changes to complex clinical decision-making processes.

Facilitation is not a magic bullet to effect science-driven health care or to realize practice and organizational performance improvements, but its connection to organizational learning is pivotal. Also pivotal is considering facilitation for inclusion in the learning strategies of health systems aspiring to become learning health systems. Facilitation relates to realizing the latent learning capacity of organizations, generating new knowledge, and overcoming normal human tendencies to take reductionist approaches to problem-solving that afford only lower-order learning.

## Additional file

Additional file 1: Table S1. This table provides different definitions of facilitation offered in the literature; Table 1a This table provides all primary references relating to elements listed in Table 1 and included in the reviews referenced in the column headings. Table 2a This table provides all primary references relating to elements listed in Table 2 and included in the reviews referenced in the column headings.
