# Supplementary material for: Why (we think) facilitation works: insights from organizational learning theory
Source: Implement Sci. 2015 Oct 6;10:141. doi: 10.1186/s13012-015-0323-0 (PMC4596304; doi:10.1186/s13012-015-0323-0)
Supplement: Additional file 1: Table S1. — This table provides different definitions of facilitation offered in the literature; Table 1a This table provides all primary references relating to elements listed in Table 1 and included in the reviews referenced in the column headings. Table 2a This table provides all primary references relating to elements listed in Table 2 and included in the reviews referenced in the column headings. [file 13012_2015_323_MOESM1_ESM.docx]

**Table S1. Definitions of Facilitation**

| “a goal-oriented dynamic process in which participants work together in an atmosphere of genuine mutual respect in order to learn through critical reflection.” [86:401] |
| --- |
| “a technique by which one person makes things easier for others…The term describes the type of support required to help people change their attitudes, habits, skills, ways of thinking, and working.” [6:152] |
| The use of “personal contact between the facilitator and the professional to encourage good practice and better service organisation.” [51:626] |
| “the process of enabling (making easier) the implementation of evidence into practice…Facilitation is achieved by an individual carrying out a specific role (a facilitator), which aims to help others.” [4:579]. |
| “involves helping others to identify questions of practice; providing support to enable others to meet specific goals, including research use; attending to the process of achieving those goals; and knowing the system in which change is proposed and implemented.” [87:325] |
| “a deliberate and valued process of *interactive problem solving* and *support* that occurs in the context of a recognized need for improvement and a supportive interpersonal relationship. Facilitation is primarily a distinct role with a number of potentially crucial behaviors and activities.” [56] |
| “guiding, coaching, mentoring, cheerleading, and encouraging.” [88:6] |
| “the process of providing support to individuals or groups to achieve beneficial change…It has been described as ‘the provision of opportunity, resources, encouragement and support for the group to succeed in achieving its own objectives and to do this through enabling the group to take control and responsibility for the way they proceed.” [69:38] |
| “a mechanism or intervention for the implementation of evidence into practice. A facilitator is an individual who is skilled in working with the concepts of change management and individual and organisational development.” [89:2] |

**Table 1a. Map of Facilitation Processes & Activities to *External* Absorptive Capacity Meta-Routines**

| ***External*** **Absorptive Capacity Meta-Routines** [9] | **Facilitation Processes & Activities**  [1, 47] | **Primary Sources** |
| --- | --- | --- |
| Identifying & recognizing the value of externally generated knowledge | Introduces new research-based ideas of potential value to resolving performance gaps | [6, 46, 51, 55, 56, 59, 90-92, 121] |
| Learning from & with partners, suppliers, customers, competitors, and consultants | Establishes effective communication channels | [46, 56, 63] |
|  | Networking | [136-138] |
|  | Supports the development of new competencies or skills by identifying external suppliers | [56] |
| Transferring knowledge back to the organization (establishing knowledge sharing processes) | Establishes effective communication channels | [136-138] |

**Table 2a. Map of Facilitation Processes & Activities to *Internal* Absorptive Capacity Meta-Routines**

| ***Internal*** **Absorptive Capacity Meta-Routines** [9] | **Facilitation Processes & Activities**  [1, 47] | **Primary Sources** |
| --- | --- | --- |
| Facilitating variation | Encourages critical assessment of current practice that leads to identification of performance gap(s) | [6, 51, 55, 56, 90-93] |
|  | Introduces new ideas (i.e., research and associated knowledge that may address performance gaps) | [59] |
|  | Enhances staff receptivity to change | [57, 58, 94, 95] |
|  | Identifies resources needed to support change | [51, 56, 60–62, 96] |
|  | Motivates and encourages others to make a change | [61, 62, 65, 97-101] |
|  | Supports the development of new competencies/skills among staff | [97, 102-111] |
| Managing internal selection regimes | Assists in establishing common goals | [4, 46, 56, 98, 112] |
|  | Enables implementation of evidence into practice  Enables research use | [4, 6, 46, 47, 95, 113, 114] |
|  | Attends to the process of achieving goals | [4, 46, 56, 98, 112] |
|  | Provides feedback about research use | [56, 115] |
| Sharing knowledge & superior practices across the organization | Establishes effective (internal) communication channels | [46, 56, 63, 116] |
|  | Promotes a culture for change  Creates a supportive (local) climate  Creates a vision that embraces evidence-based practice | [4, 58, 59, 67, 68, 92, 96, 100, 104, 117-124] |
| Reflecting, updating, & replicating  (retention) | Tailors facilitation activities to local needs and circumstances | [46, 108, 113, 114, 116, 125] |
|  | Provides ongoing support & resources to achieve goals | [46, 56, 98, 112] |
|  | Facilitates trialable initiatives | [53, 107, 124, 126-133] |
|  | Maintains change momentum | [61, 62, 65, 97-101] |
|  | Supports the development of new competencies/skills among staff | [61, 62, 65, 97-101] |
|  | Supports a goal-oriented dynamic process that promotes learning through critical reflection | [5, 56] |
| Managing adaptive tension (continuous progression) | Creates a vision that embraces evidence-based practice | [123, 124] |
|  | Promotes a culture for change  Creates a supportive (local) climate | [123, 124] |
|  | Empowers staff | [63, 131, 135] |

**Additional References for Tables S1, 1a and 2a**

(continued from references for main text)

[86] Burrows D. Facilitation: a concept analysis. Journal of Advanced Nursing. 1997;25(2):396-404.

[87] Ferguson L, Milner M, Snelgrove-Clarke E. The role of intermediaries: getting evidence into practice. Journal of Wound Ostomy & Continence Nursing. 2004;31(6), 325-327.

[88] Robertson J. Coaching leadership learning through partnership. School Leadership and Management. 2009;29(1), 39-49.

[89] Seers K, Cox K, Crichton NJ, Edwards RT, Eldh AC, Estabrooks CA,…Wallin L. FIRE (Facilitating Implementation of Research Evidence): a study protocol. Implementation Science. 2012;7(1):25.

[90] Bayley MT, Hurdowar A, Richards CL, et al. Barriers to implementation of stroke rehabilitation evidence: findings from a multi-site pilot project. Disabil Rehabil. 2012;34(19):1633-1638. doi: 10.3109/09638288.2012.656790 [published Online First: Epub Date].

[91] Borbas C, Morris N, McLaughlin B, Asinger R, Gobel F. The role of clinical opinion leaders in guideline implementation and quality improvement. Chest. 2000;118(2 SUPPL.):24S-32S.

[92] Liddy C, Laferriere D, Baskerville B, Dahrouge S, Knox L, Hogg W. An overview of practice facilitation programs in Canada: current perspectives and future directions. Healthc Policy. 2013;8(3):58-67.

[93] Pepler CJ, Edgar L, Frisch S, et al. Strategies to increase research-based practice: interplay with unit culture. Clinical Nurse Specialist. 2006;20(1):23-31.

[94] Ervin NE. Clinical coaching: a strategy for enhancing evidence-based nursing practice. Clinical Nurse Specialist. 2005;19(6):296-301.

[95] Kitson AL, Rycroft-Malone J, Harvey G, McCormack B, Seers K, Titchen A. Evaluating the successful implementation of evidence into practice using the PARiHS framework: theoretical and practical challenges. Implement Sci. 2008;3:1.

[96] Aitken LM, Hackwood B, Crouch S, et al. Creating an environment to implement and sustain evidence based practice: a developmental process. Aust Crit Care. 2011;24(4):244-254.

[97] Buonocore D. Leadership in action: creating a change in practice. AACN Clin Issues. 2004;15(2):170-181.

[98] Byron S, Moriarty D, O'Hara A. Macmillan nurse facilitators: establishing a palliative resource nurse network in primary care. Int J Palliat Nurs. 2007;13(9):438-444.

[99] Dilworth, S., Higgins, I., Parker, V., Kelly, B., & Turner, J. Exploring the situational complexities associated practice change in health. Qualitative Research Journal. 2013;13(2), 178-186.

[100] Muller A, McCauley K, Harrington P, Jablonski J, Strauss R. Evidence-based practice implementation strategy: the central role of the clinical nurse specialist. Nurs Adm Q. 2011;35(2):140-151.

[101] Vaughan C, Reddy P, Dunbar J. From rural beginnings to statewide roll-out: evaluation of facilitator training for a group-based diabetes prevention program. Aust J Rural Health. 2010;18(2):59-65.

[102] English M, Nzinga J, Mbindyo P, Ayieko P, Irimu G, Mbaabu L. Explaining the effects of a multifaceted intervention to improve inpatient care in rural Kenyan hospitals--interpretation based on retrospective examination of data from participant observation, quantitative and qualitative studies. Implement Sci. 2011;6:124.

[103] Gerrish K, McDonnell A, Nolan M, Guillaume L, Kirshbaum M, Tod A. The role of advanced practice nurses in knowledge brokering as a means of promoting evidence-based practice among clinical nurses. J Adv Nurs. 2011;67(9):2004-2014.

[104] Gerrish K, Nolan M, McDonnell A, Tod A, Kirshbaum M, Guillaume L. Factors influencing advanced practice nurses' ability to promote evidence-based practice among frontline nurses. Worldviews Evid Based Nurs. 2012;9(1):30-39.

[105] Linnebur SA, Fish DN, Ruscin JM, et al. Impact of a multidisciplinary intervention on antibiotic use for nursing home-acquired pneumonia. Am J Geriatr Pharmacother. 2011;9(6):442-450.

[106] Moriarty D, O'Hara A, Byron S. Macmillan nurse facilitators for palliative care: evaluation of a pilot project. Int J Palliat Nurs. 2007;13(7):334-343.

[107] Pannucci CJ, Jaber RM, Zumsteg JM, Golgotiu V, Spratke LM, Wilkins EG. Changing practice: implementation of a venous thromboembolism prophylaxis protocol at an academic medical center. Plast Reconstr Surg. 2011;128(5):1085-1092.

[108] Ragazzi H, Keller A, Ehrensberger R, Irani A-M. Evaluation of a practice-based intervention to improve the management of pediatric asthma. J Urban Health. 2011;88(Suppl 1):38-48.

[109] Rugh JD, Sever N, Glass BJ, Matteson SR. Transferring evidence-based information from dental school to practitioners: a pilot "academic detailing" program involving dental students. J Dent Educ. 2011;75(10):1316-1322.

[110] Rycroft-Malone J, Seers K, Chandler J, et al. The role of evidence, context, and facilitation in an implementation trial: implications for the development of the PARIHS framework. Implement Sci. 2013;8:28. doi: 10.1186/1748-5908-8-28 [published Online First: Epub Date].

[111] Rycroft-Malone J, Seers K, Crichton N, et al. A pragmatic cluster randomised trial evaluating three implementation interventions. Implement Science. 2012;7:80.

[112] Stetler CB, Damschroder LJ, Helfrich CD, Hagedorn HJ. A guide for applying a revised version of the PARIHS framework for implementation. Implement Sci. 2011;6:99. doi: 10.1186/1748-5908-6-99 [published Online First: Epub Date].

[113] Ellis I, Howard P, Larson A, Robertson J. From workshop to work practice: an exploration of context and facilitation in the development of evidence-based practice. Worldviews Evid Based Nurs. 2005;2(2):84-93. doi: 10.1111/j.1741-6787.2005.04088.x [published Online First: Epub Date].

[114] Graham ID, Harrison MB, Brouwers M, Davies BL, Dunn S. Facilitating the use of evidence in practice: evaluating and adapting clinical practice guidelines for local use by health care organizations. J Obstet Gynecol Neonatal Nurs. 2002;31(5):599-611.

[115] Wallin L, Rudberg A, Gunningberg L. Staff experiences in implementing guidelines for Kangaroo Mother Care--a qualitative study. International Journal of Nursing Studies. 2005;42:61-73.

[116] Harrison MB, Graham ID, van den Hoek J, Dogherty EJ, Carley ME, Angus V. Guideline adaptation and implementation planning: a prospective observational study. Implement Sci. 2013;8:49. doi: 10.1186/1748-5908-8-49 [published Online First: Epub Date].

[117] Byng R, Jones R, Leese M, Hamilton B, McCrone P, Craig T. Exploratory cluster randomised controlled trial of shared care development for long-term mental illness. Br J Gen Pract. 2004;54(501):259-266.

[118] Christl B, Lloyd J, Krastev Y, Litt J, Harris MF. Preventing vascular disease - effective strategies for implementing guidelines in general practice. Aust Fam Physician. 2011;40(10):825-828.

[119] Clarkson JE, Bonetti D. Why be an evidence-based dentistry champion? J Evid Based Dent Pract. 2009;9(3):145-150.

[120] Harrison LL, Kitchens EK. Implementing the research facilitator role. Nurse Educ. 1989;14(5):21-26.

[121] Harvey G, Fitzgerald L, Fielden S, et al. The NIHR collaboration for leadership in applied health research and care (CLAHRC) for greater Manchester: combining empirical, theoretical and experiential evidence to design and evaluate a large-scale implementation strategy. Implement Sci. 2011;6:96. doi: 10.1186/1748-5908-6-96 [published Online First: Epub Date].

[122] Jamerson PA, Vermeersch P. The role of the nurse research facilitator in building research capacity in the clinical setting. J Nurs Adm. 2012;42(1):21-27.

[123] Locock L, Dopson S, Chambers D, Gabbay J. Understanding the role of opinion leaders in improving clinical effectiveness. Soc Sci Med. 2001;53(6):745-757. doi: 10.1016/S0277-9536%2800%2900387-7 [published Online First: Epub Date].

[124] Ploeg J, Davies B, Edwards N, Gifford W, Miller PE. Factors influencing best-practice guideline implementation: lessons learned from administrators, nursing staff, and project leaders. Worldviews Evid Based Nurs. 2007;4(4):210-219. doi: 10.1111/j.1741-6787.2007.00106.x [published Online First: Epub Date].

[125] Åberg AC, Lundin-Olsson L, Rosendahl E. Implementation of evidence-based prevention of falls in rehabilitation units: a staff's interactive approach. J Rehabil Med 2009;41(13):1034-1040.

[126] Edwards A, Rhydderch M, Engels Y, et al. Assessing organisational development in European primary care using a group-based method. Int J Health Care Qual Assur. 2010;23(1):8.

[127] Fineout-Overholt E, Levin RF, Melnyk BM. Strategies for advancing evidence-based practice in clinical settings. J N Y State Nurses Assoc. 2004;35(2):28-32.

[128] Foley KL, Pockey JR, Helme DW, et al. Integrating evidence-based tobacco cessation interventions in free medical clinics: opportunities and challenges. Health Promot Pract. 2012;13(5):687-695.

[129] Hogg W, Lemelin J, Graham I, et al. Improving prevention in primary care: evaluating the effectiveness of outreach facilitation. Fam Pract. 2008;25(1):40-48. doi: 10.1093/fampra/cmm070 [published Online First: Epub Date].

[130] Hulscher ME, van Drenth BB, van der Wouden JC, Mokkink HG, van Weel C, Grol RP. Changing preventive practice: a controlled trial on the effects of outreach visits to organise prevention of cardiovascular disease. Qual Health Care. 1997;6(1):19-24.

[131] Pattinson RC, Arsalo I, Bergh AM, Malan AF, Patrick M, Phillips N. Implementation of kangaroo mother care: a randomized trial of two outreach strategies. Acta Paediatr. 2005;94(7):924-7 doi: 10.1080/08035250510028399 [published Online First: Epub Date].

[132] Sullivan G, Duan N, Mukherjee S, Kirchner J, Perry D, Henderson K. The role of services researchers in facilitating intervention research. Psychiatr Serv. 2005;56(5):537-542. doi: 10.1176/appi.ps.56.5.537 [published Online First: Epub Date].

[133] Wiechula R, Kitson A, Marcoionni D, Page T, Zeitz K, Silverston H. Improving the fundamentals of care for older people in the acute hospital setting: facilitating practice improvement using a Knowledge Translation Toolkit. Int J Evid Based Healthc. 2009;7(4):283-295. doi: 10.1111/j.1744-1609.2009.00145.x [published Online First: Epub Date].

[134] Robinson J. Improving practice through a system of clinical supervision. Nurs Times. 2005;101(23):30-32.

[135] Wilkinson JE, Nutley SM, Davies HTO. An exploration of the roles of nurse managers in evidence-based practice implementation. Worldviews Evid Based Nurs. 2011;8(4):236-246. doi: 10.1111/j.1741-6787.2011.00225.x [published Online First: Epub Date].

[136] Flodgren G, Parmelli E, Doumit G, et al. Local opinion leaders: effects on professional practice and health care outcomes. Cochrane Database Syst Rev. 2011;8:CD000125.

[137] Frantsve-Hawley J, Meyer DM. The evidence-based dentistry champions: a grassroots approach to the implementation of EBD. J Evid Based Dent Pract. 2008;8(2):64-69.

[138] Russell-Babin KA. Calling all opinion leaders! Keys to the diffusion of evidence. Nurs Manage. 2010;41(9):8-11.
